# Seasonal variability does not impact *in vitro* fertilization success

**DOI:** 10.1038/s41598-019-53919-3

**Published:** 2019-11-20

**Authors:** Xitong Liu, Haiyan Bai, Ben W. Mol, Wenhao Shi, Ming Gao, Juanzi Shi

**Affiliations:** 1 Assisted Reproduction Center, Northwest Women’s and Children’s Hospital, Xi’an, China; 2 Department of Obstetrics and Gynaecology, Monash University, Clayton, Australia

**Keywords:** Genetics, Risk factors

## Abstract

It is unknown whether seasonal variation influences the outcome of *in vitro* fertilization (IVF). Previous studies related to seasonal variation of IVF were all small sample size, and the results were conflicting. We performed a retrospective cohort study evaluating the relationship between seasonal variability and live birth rate in the year of 2014–2017. Patients were grouped into four seasons (Winter (December-February), Spring (March-May), Summer (June-August), and Autumn (September-November)) according to the day of oocyte pick-up (OPU). Multivariate logistic regression analysis was performed to evaluate association between seasonal variation and live birth. Models were adjusted for covariates including temperature, sunshine hour, infertility type, infertility duration, infertility factor and BMI. In total 38,476 women were enrolled, of which 25,097 underwent fresh cycles, 13,379 were frozen embryo transfer. Live birth rates of fresh embryo transfer were 50.36%, 53.14%, 51.94% and 51.33% for spring, summer, autumn and winter, respectively. Clinical pregnancy rate between the calendar months varied between 55.1% and 63.4% in fresh embryo transfer (ET) and between 58.8% and 65.1% in frozen embryo transfer (FET) (*P*-values 0.073 and 0.220). In the unadjusted model and adjust model, seasonal variation was not associated with live birth. In conclusion, there was no significant difference of seasonal variations in the outcome of IVF with fresh embryo transfer and frozen embryo transfer.

## Introduction

Several epidemiologic studies have demonstrated seasonal changes in natural pregnancy and birth rate^1,2^. However, sexual activity in humans is not bound to seasons. The impact of seasonal variability on outcome of IVF has been studied in small studies, and conflicting results have been published^3,4^. Some studies categorized patients by seasons, some by calendar months. One retrospective study did not demonstrate any significant influence of the calendar months or seasons on the IVF outcome of fresh or frozen embryo transfers. However, the outcome of the study was clinical pregnancy rate and did not adjust for covariates^5^. However, one retrospective observational cohort study indicated seasonality have a significant influence on the fertilization process and on the quality of the human embryos that are obtained *in vitro*^6^. To date, no study reported on live birth rate or birth weight. What’s more, data from China is limited.

We wanted to answer the question whether seasonal variation has impact on IVF outcome. We therefore performed a large observational study to assess a possible relationship on the seasonal influence on IVF outcome.

## Materials and Methods

This retrospective study was performed in the Assisted Reproduction Center, Northwest Women’s and Children’s Hospital in Xi’an, Shaanxi province, China and was approved by the ethics committee of the Northwest Women’s and Children’s Hospital (number 2018002). Because of the nature of retrospective cohort study, informed consent was therefore waived. We confirm that all experiments were performed in accordance with relevant guidelines and regulations. A total of 38476 patients undergoing IVF/FET between 2014 and 2017 were included in this study. All the patients had signed informed consent of IVF/FET procedure. Data was extracted from electronic medical record system.

We studied both IVF and ICSI cycles, as well as fresh and frozen transfers. We applied different protocols for COH in fresh embryo transfer cycles. Recombinant FSH or urinary FSH and/or human menopausal gonadotropins were used with daily doses between 100 and 450 IU according to patients’ age, basal FSH and antral follicle count (AFC) and could be adjusted according to ovarian response. Follicle growth was monitored by transvaginal ultrasound and hormone level was tested every other day after the follicle diameter reached 14 mm. We administrated 5,000–10,000 units of human chorionic gonadotrophin (hCG) or recombinant hCG trigger when at least three leading follicles reached a diameter of 17 mm. Oocytes were retrieved 34–36 h later. Conventional IVF or ICSI was performed after oocyte retrieval. The presence of two pronuclei (2PN) were observed 16–18 h later as the mark of fertilization. Embryos were cultured in G5-medium (Vitrolife, Sweden). Embryo scoring was performed by the combination of blastomere number, size, and fragmentation^7^. Three to five days after oocyte retrieval 1 or 2 good quality embryos were transferred into the uterus.

Our IVF center is in Xi’an, Shaanxi province of China. It is dominated by monsoon climate of medium latitudes, with high temperature and rain in summer and cold dry climate in winter, which gives us a perfect model to study the relationship of seasonal variation and IVF outcome. Monthly weather conditions (°C) and sunshine hours (Hrs) were extracted from the website of Shaanxi Provincial Bureau of Statistics. Meteorological data of Shaanxi province (China) during the year of 2014–2017 were shown in Supplementary Table 1.

The inclusion criteria were women who completed embryo transfer either in fresh cycle or frozen cycle. The exclusion criteria were: (1). history of recurrent pregnancy loss, (2). uterine pathology, (3) cycles that were cancelled due to no embryo available or failure of embryo thaw survival.

Among the 25097 IVF/ICSI stimulation cycles, 13,223(52.69%) cycles reached fresh embryo transfer. The other cycles embryos were frozen due to high risk of ovarian hyperstimulation syndrome (OHSS) (n = 2969), serum progesterone level >5.72 nmol/L (n = 832), no embryo available (n = 119), PGT (n = 1188), abnormal endometrium (n = 1782), patients with upper respiratory tract infection (n = 232) or diarrhea (n = 118) and other conditions (n = 4634) on the day of ET. Intramuscular injection of 60 mg Progesterone or 600 mg of vaginal micronized progesterone per day was given from the day of embryo transfer. The calendar months of embryo transfer were grouped to seasons, and each season lasting 3 months: spring (March–May), summer (June–August), fall (September–November) and winter (December–February).

### Definition of clinical outcomes

Clinical pregnancy was defined as one or more gestational sacs confirmed by ultrasound. Miscarriage was defined as spontaneous loss of a clinical pregnancy before 22 completed weeks of gestational age. Ectopic pregnancy was defined as gestational sac observed by ultrasound outside the uterine cavity. Live birth was defined as the number of deliveries that resulted in at least one live birth >24 weeks.

The descriptive data on participants’ characteristics were summarized using the mean and standard deviation for continuous variables. Counts and proportions were used for the categorical variables. Chi-squared tests were performed to compare the categorical variables. For multivariate analyses, logistic regression was utilized. Crude odds ratios (OR) and adjusted ORs (AOR) with 95% confidence interval (CI) were calculated to assess the association between covariates and live birth. In the adjusted Model I: we adjusted for temperature and sunshine hour. In the adjusted Model II: we adjusted for temperature, sunshine hour, infertility type, infertility duration, infertility factor and BMI. All of the analyses were performed with SPSS Version 13.0 (Statistical Package for Social Science, Inc., Chicago, IL, USA). The level of significance was set at *p* < 0.05.

## Results

We included 25097 ovarian stimulation and OPU cycles of which 13223 cycles resulted in fresh embryo transfer and 13379 frozen embryo transfer cycles. The comparison of the general information among seasonal groups is shown in Table 1. In summer, the mean age of the patients was youngest (31.10 ± 5.07). BMI (22.49 ± 3.27) was highest and duration of infertility (3.96 ± 3.03) was longest in spring. Infertility type did not differ among seasonal groups. Tubal factor accounts for the largest proportion in summer (41.52%). In summer, patients were allocated agonist protocol most (81.80%). Male age was oldest in spring (33.13 ± 5.82), while sperm concentration was highest in autumn (54.97 ± 274.44) with significant difference.

**Table 1:** Comparison of the general information among seasonal groups in fresh embryo transfer.

	Spring (Mar–May)	Summer (Jun–Aug)	Autumn (Sep–Nov)	Winter (Dec–Feb)	Total	*P* value
No. of patients	6566	7192	6124	5215	25097	
Female age(years)	31.32 ± 5.29	31.10 ± 5.07	31.32 ± 5.26	31.37 ± 5.30	31.27 ± 5.22	0.017
BMI (kg/m^2^)	22.49 ± 3.27	22.34 ± 3.24	22.29 ± 3.22	22.39 ± 3.18	22.38 ± 3.23	0.005
Infertility duration(years)	3.96 ± 3.03	3.82 ± 2.96	3.67 ± 2.94	3.67 ± 3.00	3.79 ± 2.98	< 0.001
Infertility type						0.433
rimary infertility (%)	3414(52.00)	3837(53.40)	3245(53.00)	2742(52.60)	13238(52.70)	
Secondary infertility (%)	3152(48.00)	3355(46.60)	2879(47.00)	2473(47.40)	11859(47.30)	
Infertility factor						0.008
Tubal factor	2688 (40.94%)	2986 (41.52%)	2371 (38.72%)	2042 (39.16%)	2688 (40.94%)	
Ovulation disorder	543 (8.27%)	611 (8.50%)	535 (8.74%)	475 (9.11%)	543 (8.27%)	
Endometriosis	91 (1.39%)	95 (1.32%)	83 (1.36%)	69 (1.32%)	91 (1.39%)	
Female mixed factor	771 (11.74%)	866 (12.04%)	823 (13.44%)	613 (11.75%)	771 (11.74%)	
Male factor	1235 (18.81%)	1287 (17.89%)	1152 (18.81%)	1030 (19.75%)	1235 (18.81%)	
Both female and male factor	987 (15.03%)	1051 (14.61%)	893 (14.58%)	748 (14.34%)	987 (15.03%)	
PGT	99 (1.51%)	89 (1.24%)	96 (1.57%)	71 (1.36%)	99 (1.51%)	
Other	152 (2.31%)	207 (2.88%)	171 (2.79%)	167 (3.20%)	152 (2.31%)	
Protocol						< 0.001
Agonist (%)	5251(80.00)	5886(81.80)	4141(67.60)	3957(75.90)	19235(76.60)	
Antagonist (%)	1222(18.60)	1126(15.70)	1072(17.50)	897(17.20)	4317(17.20)	
Other (%)	93(1.40)	180(2.50)	911(14.90)	361(6.90)	1545(6.20)	
Male age (years)	33.13 ± 5.82	32.83 ± 5.67	33.08 ± 5.84	33.10 ± 5.76	33.02 ± 5.77	0.008
Sperm concentration	49.36 ± 39.38	48.77 ± 38.24	54.97 ± 274.44	54.22 ± 153.84	51.57 ± 155.38	0.045

Abbreviation: BMI, body mass index.

We harvested a mean number of 10.47 ± 6.66 oocytes (Supplementary Table 2). In February, the number of OPU reached highest while in December, it dropped to the lowest. However, clinical pregnancy rate did not differ among 12 months. Likewise, analysis of FET cycles did not show any significant differences in the clinical pregnancy rate. In March, live birth rate seems to be lower. Thus, we compared live birth rate from the year of 2014 to 2017 and found live birth rate did not differ between different months.

IVF outcome among seasonal groups were shown in Table 2. The number of oocytes retrieved per OPU cycle and number of embryos transferred did not significantly differ between seasons. Meanwhile, gonadotropin (Gn) dosage and duration were largest in autumn. In spring and winter, the number of top-quality embryos was largest among seasons. In summer, clinical pregnancy rate reached highest, however, no difference was found in live birth rate and birth weight. Miscarriage rate and ectopic pregnancy rate also did not differ among seasons.

**Table 2:** Comparison of IVF outcome among seasonal groups in fresh cycles.

	Spring	Summer	Autumn	Winter	Total	*P*-value
Gn dosage (IU)	2424.38 ± 1043.59	2394.19 ± 1016.88	2579.04 ± 1471.53	2437.53 ± 1088.58	2456.25 ± 1166.71	< 0.001
Gn duration(days)	10.29 ± 2.80	10.44 ± 2.92	10.46 ± 2.81	10.37 ± 2.97	10.39 ± 2.88	0.003
No. of OPU cycles	6537	7160	6088	5192	24977	
No. of oocytes retrieved per OPU cycle	10.61 ± 6.78	10.47 ± 6.50	10.36 ± 6.66	10.40 ± 6.71	10.47 ± 6.66	0.180
cleavage rate (%)	98.19	98.44	97.81	97.88	98.11	0.033
No. of top-quality embryos	2.98 ± 2.95	2.90 ± 2.76	2.69 ± 2.86	2.98 ± 3.01	2.89 ± 2.90	0.001
No. of embryo transferred per cycle	1.70 ± 0.49	1.70 ± 0.48	1.69 ± 0.48	1.69 ± 0.48	1.69 ± 0.48	0.50
Clinical pregnancy rate per embryo transfer (%)	2011/3431(58.60)	2387/3850(62.00)	1897/3125(60.70)	1685/2817(59.80)	7980/13223(60.30)	0.027
Miscarriage rate (%)	250/2011(12.40)	307/2387(12.90)	244/1897(12.90)	211/1685(12.50)	1012/7980	0.985
Ectopic pregnancy rate	24/2011(1.20)	31/2387(1.30)	27/1897(1.40)	25/1685(1.50)	107/7980(1.30)	0.867
Live birth rate (%)	1728/3431(50.36)	2046/3850(53.14)	1623/3125(51.94)	1446/2817(51.33)	6843/13223(51.75)	0.118
Birth weight	3.09 ± 0.59	3.10 ± 0.58	3.11 ± 0.59	3.07 ± 0.59	3.10 ± 0.59	0.152

A logistic regression model was then used to assess the association between seasonal variation and live birth while adjusting for potential confounders presented in Table 3. In the unadjusted model, seasonal variation was not associated with live birth. After adjusting for covariates, the association partially disappear, which indicated seasonal variation may impact live birth rate in IVF. In addition, the effect of winter is independent of sunshine and temperature.

**Table 3:** Relationship between seasonal variation and live birth in different models.

Outcome	Crude Model		Adjusted Model I		Adjusted Model II	
	OR (95% CI)	*P*-value	OR (95% CI)	*P*-value	OR (95% CI)	*P*-value
Season						
Spring	Reference		Reference		Reference	
Summer	1.11 (1.03, 1.20)	0.005	1.05 (0.94, 1.17)	0.389	1.05 (0.94, 1.17)	0.408
Autumn	1.01 (0.93, 1.09)	0.813	1.04 (0.94, 1.15)	0.456	1.04 (0.94, 1.16)	0.431
Winter	1.07 (0.99, 1.17)	0.087	1.08 (1.02, 1.11)	0.065	1.09 (1.00, 1.21)	0.059

OR, odds ratio. CI, confidence interval.

Crude model: we did not adjust for other covariates.

Adjusted Model I: we adjusted for temperature and sunshine hour.

Adjusted Model II: we adjusted for temperature, sunshine hour, infertility type, infertility duration, infertility factor and BMI.

## Discussion

The seasonal variability of fertility has been studied in animals. However, seasonal influence on outcome of IVF in human is still under discussion. The effect of seasonal change on natural conception mainly in two ways: weather condition and temperature affect male sperm quality^8^, sunshine exposure influence female’s ovulation. The optimal spermatogenesis in man requires 2–6°C lower testicular temperature than body and any rise above physiological temperature of the testes has adverse effect on spermatogenesis^9,10^. IVF procedure exert an extensive control over temperature, moisture, light and other condition, and huge effort has been made to standardize protocols and laboratory procedures and minimize any external influence on IVF outcome. Nevertheless, the effect of seasonal variability on IVF has been reported by some articles^6,11^, while others have reached different conclusions^12^. The inconsistent results are attributed to different inclusion criteria, widely diverse environment with different temperature and sunlight exposure when studies were conducted.

In this study, we analyzed the seasonal variation of different variables related to IVF outcome. We use 3-month seasonal periods to minimize the cycle uneven distribution. Our results showed no significant differences in the number of oocytes retrieved, live birth rate or birth weight. Our findings were consistent with some studies^13^. One hypothesis is that in summer, male sperm quality is affected by temperature. Our data confirmed this hypothesis showing that male sperm concentration was lowest in summer with significant difference. However, no difference was found in clinical pregnancy rate among months and live birth rate among seasons. One explanation is that sperm concentration could be compensated by utilizing a concentrated amount of motile sperm in IVF or changing to ICSI for fertilization. However, our data from logistic regression showed seasonal variation may affect live birth rate especially in winter compared with spring. One cross-sectional study in China found lower serum vitamin D levels were associated with clinical pregnancy and live birth rate following IVF^14^. Serum 25(OH)-D levels were lowest in spring compared with other seasons, which could in turn affect pregnancy outcomes. Studies have demonstrated the fact that seasonal variation in serum AMH correlated with changes in seasonal 25(OH)-D (being 18% lower in winter than in summer)^15^.

There are other methods for controlling for time collinearity. A time-series study could be performed to assess the association of seasonal variation and live birth rate and evaluating the degree of the effects on estimation. In addition, sensitivity analyses can be conducted to examine whether there were nonlinear association between months and live birth rate.

This study showed there was a trend of decreasing clinical pregnancy in February and March, but it did not reach significant difference. One explanation of monthly fluctuations is the climate change with sunlight exposure, a direct melatonin or neurotransmitter effect on the end-organ^16,17^. One study from Finland suggested melatonin secretion is increased during the dark season (November-January) when the amount of daylight is about 3–4 h, while ovarian activity is decreased^12^. Data from animal studies indicate the length of the nocturnal melatonin pulse is of central importance in gonadal regulation^18^.

In conclusion, by the analysis of the largest sample size to date, we confirm there was no significant difference of seasonal variations in the outcome of IVF with fresh embryo transfer and frozen embryo transfer. A routine IVF treatment should not be changed by season.

## Supplementary information

supplementary table
