# Supplementary material for: Seasonal variability does not impact in vitro fertilization success
Source: Sci Rep. 2019 Nov 20;9:17185. doi: 10.1038/s41598-019-53919-3 (PMC6868277; doi:10.1038/s41598-019-53919-3)
Supplement: Supplementary file 1 — supplementary table [file 41598_2019_53919_MOESM1_ESM.pdf]

**Title: Seasonal variability does not impact in vitro fertilization success**

Xitong Liu, MD[1], Haiyan Bai, MD[1], Ben W. Mol[2], Wenhao Shi, MD[1], Ming Gao, MD[1], Juanzi Shi, PhD[1]
[1]Assisted Reproduction Center, Northwest Women's and Children's Hospital, Xi'an, China
[2]Department of Obstetrics and Gynaecology, Monash University, Clayton, Australia

Supplementary table 1. Meteorological data of Shaanxi province (China) during the year of 2014-2017.

| | Temperature (℃) | | | | Sunshine hour (Hrs) | | | |
|---|---|---|---|---|---|---|---|---|
| | 2014 | 2015 | 2016 | 2017 | 2014 | 2015 | 2016 | 2017 |
| January | 2.9 | 2.3 | 0.3 | 2.7 | 181.5 | 127.0 | 111.9 | 106.9 |
| February | 2.3 | 5.6 | 4.8 | 5.6 | 55.4 | 127.1 | 205.5 | 162.7 |
| March | 12.0 | 10.5 | 11.5 | 9.8 | 162.5 | 144.3 | 168.8 | 161.8 |
| April | 16.1 | 16.3 | 18.3 | 17.0 | 126.0 | 214.3 | 211.9 | 195.9 |
| May | 21.1 | 21.4 | 20.2 | 22.2 | 209.3 | 200.4 | 200.7 | 232.2 |
| June | 26.0 | 24.3 | 26.7 | 26.1 | 193.9 | 116.6 | 258.9 | 216.5 |
| July | 29.1 | 28.1 | 28.3 | 30.5 | 288 | 257.1 | 231.6 | 290.9 |
| August | 25.1 | 26.0 | 28.6 | 26.6 | 191.4 | 208.5 | 290.7 | 180.1 |
| September | 20.3 | 21.7 | 22.6 | 21.3 | 112.0 | 119.8 | 141.5 | 103.8 |
| October | 16.4 | 15.0 | 15.3 | 13.7 | 105.0 | 95.0 | 89.2 | 34.3 |
| November | 8.9 | 8.2 | 8.3 | 8.8 | 14.0 | 58.5 | 119.4 | 121.8 |
| December | 2.2 | 3.1 | 4.7 | 3.0 | 175.8 | 127.3 | 110.2 | 158.8 |
| Average | 15.2 | 15.2 | 15.8 | 15.6 | 1941.8 | 1795.9 | 2140.3 | 1965.7 |

Supplementary table 2. Distribution of treatment cycles and pregnancy rates per embryo transfer during 2014-2017.

| | Fresh embryo transfer cycles | | | | Frozen embryo transfer cycles | | | |
|---|---|---|---|---|---|---|---|---|
| | No. of OPU | No. of fresh ET | CPR (%) | LBR (%) | No. of FET cycles | No. of transferred embryos | CPR (%) | LBR (%) |
| January | 10.70±6.89 | 926 | 551/926(59.5） | 456/926(49.24) | 952 | 1.75±0.48 | 620/952(65.10) | 500/952(52.52) |
| February | 10.94±6.93 | 714 | 420/714(58.8) | 362/714(50.70) | 648 | 1.77±0.47 | 402/648(62.00) | 341/648(52.62) |
| March | 10.63±6.90 | 794 | 438/795(55.1） | 376/795(47.30) | 949 | 1.76±0.47 | 566/949(59.60) | 449/949(47.31) |
| April | 10.71±6.97 | 1206 | 731/1206(60.6) | 625/1206(51.82) | 1000 | 1.76±0.46 | 633/1000(63.30) | 512/1000(51.20) |
| May | 10.50±6.55 | 1430 | 842/1430(58.9) | 727/1430(50.84) | 1058 | 1.74±0.47 | 655/1058(61.90) | 520/1058(49.15) |
| June | 10.76±6.68 | 1360 | 841/1360(61.8) | 721/1360(53.01) | 1191 | 1.79±0.45 | 730/1191(61.30) | 598/1191(50.21) |
| July | 10.20±6.34 | 1337 | 847/1337(63.4) | 720/1337(53.85) | 1319 | 1.76±0.46 | 802/1319(60.8) | 660/1319(50.04) |
| August | 10.43±6.45 | 1153 | 699/1153(60.6) | 605/1153(52.47) | 1295 | 1.75±0.46 | 787/1294(60.80) | 648/1294(50.08) |
| September | 10.23±6.59 | 1060 | 645/1060(60.8) | 543/1060(51.23) | 1306 | 1.75±0.46 | 824/1306(63.10) | 674/1306(51.61) |
| October | 10.44±6.59 | 1096 | 658/1096(60.0) | 571/1096(52.10) | 1289 | 1.74±0.47 | 782/1289(60.70) | 619/1289(48.02) |
| November | 10.42±6.81 | 969 | 594/969(61.3) | 509/969(52.53) | 1204 | 1.74±0.47 | 708/1204(58.80) | 598/1204(49.67) |
| December | 9.85±6.39 | 1177 | 714/1177(60.7) | 628/1177(53.36) | 1168 | 1.71±0.48 | 715/1168(61.20) | 608/1168(52.04) |
| Total | 10.47±6.66 | 13223 | 7980/13223(60.3) | 6843/13223(51.75) | 13379 | 1.75±0.47 | 8224/13378(61.50) | 6718/13379(50.21) |
| *P* value | <0.001 | | 0.073 | 0.205 | | 0.024 | 0.220 | 0.261 |

CPR, clinical pregnancy rate; LBR, live birth rate
